# Heart Team - The Reality?

**DOI:** 10.21470/1678-9741-2018-0608

**Published:** 2018

**Authors:** O.P. Yadava

**Affiliations:** 1 CEO & Chief Cardiac Surgeon - National Heart Institute, New Delhi, India.

Heart Team seems to be a-la-mode for not only decision-making in Cardiology and Cardiac Surgery, but now even for performance of the procedure. The concept, though originated in 1940s with the 'tumour boards', came to fruition in cardiac services only after the pivotal SYNTAX trial and was in fact given a firm footing with the development of the transcatheter technology for aortic valve replacement and hybrid cardiac interventions. With a strong evidence base in its favor, when applied with right intent, pragmatically the concept seems to be a 'Platonic' illusion in most parts of the developing world, outside the European and North American continents^[1]^.

Though it enjoys a class I (C) level of recommendation, there are a large number of practical issues in implementing the Heart Team concept. The logistics of the availability of all the constituents of the Heart Team in form of the interventional cardiologist, cardiac surgeon, clinical cardiologist, family physician and the patient - all at an anointed time, and obvious funding requirements for implementing this concept - are important bottlenecks. The fact that Medicine is at best an imprecise science, and that it is also a rapidly evolving and moving target, so that the data is not always black and white and there are multiple shades of gray applicable to any given clinical scenario, does not helps matters. Ignorance of the patient, as also his/her lack of the ability to comprehend critical issues involved in decision-making, too hampers the concept of the Heart Team. In such situations, the patient is virtually incapable to make critical decisions, and both the patient and the relatives leave the decision-making to the treating doctor, even when an honest attempt is made to involve them. In the developing parts of the world, societal personality is still subservient to the 'Master-Subject' relationship, and even under circumstances where an opportunity is offered, they refuse to emerge from it. In fact, on the very contrary, they find comfort in this provider-receiver equation, which is the very antithesis to the concept of the Heart Team. In addition, most of the institutions in the developing world are run on hierarchical basis, so 'Evidence-Based Medicine' yields and loses out to the 'Eminence-Based Medicine'. Though it may not be acknowledged in public, the verdict of the senior most person, specially the one sitting at the helm of the administrative affairs, goes unchallenged and prevails. To bring up the rear, it is a no brainer that, with the ulterior fiscal interests of corporatization of Medicine, the poor 'medical' therapy would hardly have a chance to stand up to its powerful and almighty cousin - the 'Interventional' therapies.

Certainly, I am not decrying the concept of teamwork-based approach to any facet of Medicine, or, in fact, life in generic terms, but all I am trying to bring forward is that this concept, at least at this moment in time, is more in vogue on paper than in reality. However, I have no qualms ceding ground that it needs to change and the silo-based vertical streams that we run in most fields of Medicine, should now integrate laterally, and only when that happens, will the team-based ideology evolve and progress universally. In fact, it is a vicious circle, and even the vice-versa is true. Heart Team may facilitate dispensation of holistic and more organic medical care and thus needs to be encouraged. At the peril of repetition, I re-emphasise that its not the concept, but the logistics of its implementation which are at the core of this comment. It is therefore the need of the hour that the bull is taken by the horn, and the proverbial straw that breaks the camel's back could be a dictate from the regulatory bodies, or a premandated requirement of reimbursing agencies, for every disease process to have a combined decision-making before implementation. In the developing world, self-regulation may not produce the desired results and the salutary developments may have to be mandated by regulatory and reimbursing authorities, to deliver their purported goodness to the suffering humanity.

Another mundane question that needs to be addressed is: who should head the Heart Team? The captain should be one who can take a wider holistic view of the entire patient and not one with a narrow tubular vision. Therefore, intuitively that would be an internist. However, as Mircea Cinteza quips, "Does this guy live anymore? - I am afraid not". So, pragmatically speaking, the profile of the captain should be "... that guy of middle age, who puts together the intempestive solutions of the young and the too wise solutions of the old. And he or she elaborates and applies the winning midway solution"^[2]^. And if I can add my two pence, the head of the team should have the where-withal to tamper and moderate the personal egos and ulterior motives of the silo-based specialists. The fast-getting extinct species of Internal Medicine specialists have the best credentials and, hopefully, should fit the bill efficiently and effectively.

Epilogue - Can we have cross pollination of ideologies and thoughts of different ethnicities and continents through regular columns in each other's journals, a kind of "Editors' Heart Team"!

Food for thought!
